# Health-system preparedness for early detection of mild cognitive impairment: primary-care evidence from a national study among general practitioners in Romania

**DOI:** 10.3389/fpubh.2026.1969120

**Published:** 2026-10-23

**Authors:** Alex-Constantin Paraschiv, Luiza Spiru, Andreea Stamate, Daniela Mănuc

**Affiliations:** 1 School of Advanced Studies of the Romanian Academy (SCOSAAR), Romanian Academy, Bucharest, Romania; 2 Carol Davila University of Medicine and Pharmacy, Bucharest, Romania; 3 Department of Geriatrics, Gerontology, and Old Age Psychiatry and Longevity Medicine, Carol Davila University of Medicine and Pharmacy, Bucharest, Romania; 4 Research Department, Ana Aslan International Foundation, Bucharest, Romania

**Keywords:** cognitive screening, general practitioners, knowledge, attitudes and practices, mild cognitive impairment, Romania

## Abstract

**Background:**

Mild cognitive impairment (MCI) is a growing public health concern in aging populations. In Romania, adults aged 65 years and over comprise about one fifth of the population, and estimated MCI prevalence in this age group is among the highest in Europe (28.5%), yet no organised national programme for systematic early detection exists. General practitioners (GPs) are well positioned to contribute to population-level detection, but their knowledge, attitudes and practices regarding MCI have not been evaluated nationally. This study assessed these domains as primary-care indicators of health-system preparedness for early MCI detection.

**Methods:**

We conducted a national cross-sectional study among GPs practicing in Romania between June 2025 and January 2026, using an online self-administered questionnaire adapted from a published knowledge, attitudes and practices instrument. Analyses included descriptive statistics, Spearman correlations, group comparisons and multiple linear regression.

**Results:**

A total of 547 eligible GPs were included; 365–416 completed the questionnaire sections. The mean knowledge score was 33.20 (SD = 16.37): only 6.5% identified the Petersen diagnostic criteria, 10.8% recognized the most used cognitive screening instrument and 20.7% identified modifiable risk factors. Attitudes towards MCI detection were favorable (mean = 73.26, SD = 6.96). Nearly all GPs (97.9%) agreed that they should be involved in MCI detection, while 91.4% believed that early detection may delay progression to Alzheimer’s disease. Reported practices were more variable (mean = 73.01, SD = 11.53), and only 29.0% routinely used screening tools. Regression models explained a modest proportion of variance in reported practice. Higher practice scores were associated with more favorable attitudes, greater knowledge, MCI-specific training, and higher daily patient volume, whereas larger registered patient lists were associated with lower practice scores. Only 10.8% had received MCI-specific training.

**Conclusion:**

Romanian GPs strongly support early MCI detection, but this willingness coexists with knowledge gaps, limited screening use, scarce training and workload constraints. In a high-prevalence aging population without an organised national programme, these findings indicate a public health gap between population need and health-system preparedness. Strengthening early detection is likely to require targeted continuing medical education, feasible screening recommendations and organizational support for structured cognitive assessment.

## Background

Romania is facing a substantial burden of cognitive impairment among older adults. Recent European estimates indicate that the prevalence of mild cognitive impairment (MCI) among adults aged 65 years and older in Romania reaches 28.5%, among the highest reported values in Europe (1). This is particularly relevant in the context of population aging, as adults aged 65 years and over represent an increasing share of the Romanian population (2). Together with the growing burden of dementia and related cognitive disorders, these trends make early cognitive decline an important public health issue for patients, families and the health system (3).

MCI is generally defined as an objective cognitive decline that exceeds normal age-related changes but does not meet the diagnostic criteria for dementia and does not substantially impair daily functioning (4–6). It may affect memory, attention, language or executive function and is commonly regarded as an intermediate stage between normal cognitive aging and dementia (4, 6). Because people with MCI have an increased risk of progression to dementia, timely identification may create an opportunity for earlier clinical assessment, counselling, management of modifiable risk factors and appropriate follow-up (7). Globally, the prevalence of MCI among community-dwelling adults aged 50 years and older is estimated at 15.6% (8), while broader estimates including community, nursing home and hospital populations reach 19.7% (9); among European adults aged 65 years and over, prevalence has been estimated at 23.9% (10)—placing Romania, at 28.5%, at the upper end of reported values.

Despite its clinical and public health relevance, MCI remains frequently under-recognized in primary care. Its symptoms may be subtle, slowly progressive or interpreted by patients and families as part of normal aging. In the absence of an organised programme for systematic early detection, many cases may remain undetected until cognitive decline becomes more visible or functionally limiting (11, 12). Time constraints, limited training and the lack of clear guidance for MCI detection may further contribute to delayed recognition in routine practice (13).

General practitioners (GPs) are central to strategies aimed at improving early detection. In Romania, as in many European health systems, GPs are often the first and most regular point of contact for older adults. Their long-term relationship with patients places them in a favorable position to notice gradual cognitive, behavioral or functional changes and to initiate further assessment when needed (14, 15). However, the ability of primary care to contribute to early detection depends not only on access to patients, but also on physicians’ knowledge of MCI, attitudes toward early detection, familiarity with brief cognitive assessment tools, available consultation time, and clear guidance on how suspected cases should be recognized and referred for specialist assessment when appropriate (16).

International studies suggest that these conditions are not always met. Research conducted in China, India and several European countries has shown that physicians may support the principle of early cognitive assessment while still reporting limited training, uncertainty regarding diagnostic criteria, inconsistent use of screening instruments and practical barriers in routine care (17–19). These findings suggest that early MCI detection is not only an individual clinical task, but also a matter of primary care preparedness, service delivery and public health planning.

In Romania, previous research has examined GPs’ perceived role, current involvement and barriers in the mental health care of older adults, including memory and neurocognitive problems, Alzheimer’s disease and other neurodegenerative disorders (20). However, national evidence specifically addressing GPs’ knowledge, attitudes and practices regarding MCI detection and management is lacking. Addressing this evidence gap is particularly important given the high estimated prevalence of MCI among older adults in Romania.

Therefore, the present study aimed to assess the knowledge, attitudes and practices of GPs in Romania regarding MCI detection and management as indicators of primary care preparedness for early cognitive decline detection. The study also aimed to identify sociodemographic and professional factors associated with these domains. We hypothesised that higher knowledge and more favorable attitudes would be associated with more frequent use of reported practices related to early MCI detection. In addition, we explored whether selected professional and practice-related factors, including MCI-specific training, workload-related indicators and practice setting, were associated with GPs’ reported practices.

## Methods

### Study design and setting

We conducted a national cross-sectional study among GPs practicing in Romania. The study assessed GPs’ knowledge, attitudes and practices regarding MCI, with a focus on the role of primary care in the early detection of cognitive decline. Data were collected between 18 June 2025 and 8 January 2026 using a self-administered online questionnaire hosted on Qualtrics, disseminated through e-mail, GP groups and social media channels to reach physicians from different regions and practice settings across Romania.

### Participants and recruitment

Eligible participants were GPs currently practicing in Romania. In the Romanian primary-care system, family physicians include both physicians formally qualified in family medicine and a legacy group of general medicine physicians who graduated before 2005 and are legally assimilated to family physicians. All participants were therefore physicians currently practicing as family physicians in primary care. Family medicine residents and physicians who were retired or no longer practicing were not eligible. Participation was voluntary and anonymous. All participants provided electronic informed consent before completing the survey and could withdraw at any time without consequence.

Of 608 GPs who accessed the survey, 61 discontinued before providing consent or eligibility information, yielding 547 eligible respondents. As some participants did not complete all questionnaire sections, each analysis used the maximum number of available complete cases, and denominators are reported separately where applicable.

### Bias

Several design choices were intended to limit potential sources of bias. The questionnaire was anonymous and self-administered, which was intended to reduce social desirability pressure, although self-reported practice data remain susceptible to it. The broad dissemination across regions and both urban and rural practice settings was intended to limit selection toward any single type of practice.

### Study size

No formal *a priori* sample size calculation was performed; the questionnaire was distributed with the aim of reaching as many eligible GPs as possible at national level. Because it was disseminated through open online channels, the exact number of physicians who received the invitation could not be determined, and a conventional response rate could not be calculated.

### Measures

The questionnaire used in this study was adapted from the instrument developed by Lu et al. (17) and tailored to the Romanian context. The adaptation process involved consultation with several general practitioners (GPs) in Romania to ensure clinical relevance, clarity, and contextual appropriateness. Both the original and the Romanian versions of the instrument are provided in the Supplementary materials, and the key modifications are described below.

MCI knowledge was assessed using eight items covering prevalence, risk factors, diagnostic criteria, referral pathways, screening tools, intervention measures, prognosis, and conversion rate to dementia. Each item was scored dichotomously (1 = correct; 0 = incorrect). A total knowledge score was calculated for each respondent as the percentage of correct responses, with higher scores indicating greater knowledge of MCI.

Attitudes towards MCI were measured using 11 items assessing GPs’ perceptions of the condition, including perceived susceptibility, severity, benefits of intervention, anticipated responses from patients and society, and the role of healthcare providers. Items were rated on a five-point Likert scale ranging from 1 (strongly disagree) to 5 (strongly agree).

Items 1, 7, 8, and 9 were reverse coded to ensure that higher scores consistently reflected more favorable attitudes towards MCI management. Specifically, for these items, “strongly disagree” was coded as 5 and “strongly agree” as 1. A total attitude score was computed by dividing the sum of the item scores by the maximum possible score and multiplying by 100. The resulting score had a theoretical range of 20 to 100, with higher values indicating more favorable attitudes. A score of 60 corresponds to a neutral response across all items.

GPs’ practices in the management of MCI were assessed using an adapted version of the questionnaire developed by Lu et al. (17). The original instrument comprised 11 items. Our scale consisted of 11 items, but one item was replaced. Following consultations with specialists we added one item: “I think there is a connection between frailty and the development of mild cognitive impairment?” and eliminated the item “I would coordinate support services” as it falls outside the scope of family medicine.

The scale captures key domains of clinical practice, including detection of cognitive and psychiatric symptoms, assessment of risk factors (e.g., family history of Alzheimer’s disease), use of screening tools, referral practices, communication with patients and families.

Each item was rated on a five-point response scale (always, usually, sometimes, seldom, never), with responses coded from 5 to 1, respectively. The ‘not applicable’ option from the original scale was removed because our study targeted only family physicians, and the practices corresponding to each item are strictly within their remit. A total practice score was computed by dividing the sum of the item scores by the maximum possible score and multiplying by 100. The resulting score had a theoretical range of 20 to 100. Because the adapted items encompass reported behavior, behavioral intention and perceived importance, higher scores were interpreted as reflecting a broader composite of self-reported MCI-related practice rather than a unidimensional measure of practice frequency. A score of 60 corresponds to a “sometimes” response across all items.

Covariates included respondents’ sociodemographic characteristics and professional experience. Sociodemographic variables comprised age (<30, 30–39, 40–49, ≥50 years), gender (male, female, other), marital status (married, unmarried, other), and educational attainment (graduate, postgraduate, one specialization, more than one specialization, PhD, postdoctoral). Professional title was categorised as “primary (attending) physician,” “specialist physician,” or “no specialized fellowship.” In this classification, “primary (attending) physician” denotes the senior professional grade in the Romanian medical system, “specialist physician” refers specifically to a specialist in family medicine, and “no specialized fellowship” designates the legacy group of general medicine physicians legally assimilated to family physicians. Thus, all three categories refer to physicians practicing as family physicians and differ in professional pathway and grade rather than medical specialty.

Work-related variables included years of GP registration (<5, 5–9, 10–14, ≥15 years), number of registered patients, practice location (rural/urban), daily patient volume (<20, 21–24, 25–29, >29 visits), number of patients diagnosed with MCI, and prior MCI training (yes, no, do not know).

### Statistical analysis

Data were analysed using IBM SPSS Statistics (Version 25). Data were checked for normality and symmetry using Shapiro–Wilk tests, histograms and Q-Q plots, skewness, and Levene’s test for equality of variances (see Table 1).

**Table 1:** Skewness, kurtosis and normality tests for knowledge, attitudes and practice scores.

Variable	Skewness	SE	*Z*	Kurtosis	SE	*Z*	Shapiro–Wilk (*p*)
Knowledge score	0.40	0.12	3.36	−0.02	0.24	−0.10	< 0.001
Attitudes score	−0.10	0.13	−0.76	1.73	0.25	6.96	< 0.001
Practice score	−0.43	0.13	−3.31	0.74	0.26	2.85	< 0.001

SE = standard error. The Shapiro–Wilk test indicates statistically significant deviation from normality when *p* < 0.05.

The characteristics (categorical variables) of the study participants were described using frequency (%) distributions. Mean (M) and standard deviation (SD) values were calculated to evaluate MCI-related Knowledge, Attitudes, and Practice of the respondents in addition to frequency (%) distributions of the item responses.

The correlations between MCI-related Knowledge, Attitudes, and Practice scores, sociodemographic and professional experience variables were analysed using Spearman correlations.

MCI-related Knowledge, Attitudes, and Practice scores (dependent variables) were compared between participants with different characteristics (e.g., professional experience; level of specialization; participation in an MCI training course; sex; age group) using Kruskal-Wallis H test and Mann–Whitney U tests, respectively.

To identify factors associated with family physicians’ knowledge, attitudes, and practices (KAP) regarding mild cognitive impairment (MCI), three separate multiple linear regression analyses were performed. Each KAP domain was analysed as the dependent variable in a separate model. Independent variables included the remaining KAP domains together with demographic and professional characteristics selected based on theoretical relevance and previous literature. All models included the same set of professional and demographic predictors: number of registered patients, number of registered patients with MCI, education level, length of GP experience (years), participation in MCI training, professional title, and daily patient volume. For regression analyses, MCI training status was dichotomised as 0 = no or do not know and 1 = yes, with no/do not know as the reference category. Multi-category variables with an inherent ordering were entered as ordinal predictors using increasing category codes: education (1 = graduate to 6 = postdoctoral), professional title (0 = no specialized fellowship, 1 = specialist physician, 2 = primary [attending] physician), years of GP experience (in increasing categories), daily patient volume (1 = <20, 2 = 21–24, 3 = 25–29, 4 = > 29), number of registered patients (1 = <800, 2 = 800–1,500, 3 = 1,500–2000, 4 = 2000–2,500, 5 = > 2,500), and number of patients with MCI (1 = 1–10, 2 = 11–20, 3 = 21–30, 4 = 31–40, 5 = 41–50, 6 = > 51). Accordingly, coefficients for these ordinal predictors represent the estimated change in the outcome associated with a one-category increase.

The three KAP domains were modelled separately because knowledge, attitudes, and practices were considered conceptually distinct, although related, outcomes. This approach allowed factors associated with each domain to be examined separately while accounting for associations among the KAP domains and adjusting for the demographic and professional variables included in the models. All predictors were entered simultaneously using the Enter method. Prior to model estimation, the assumptions of multiple linear regression were evaluated. Normality of residuals was assessed through visual inspection of histograms and normal probability (P–P) plots. Linearity and homoscedasticity were examined using scatterplots of standardized residuals against standardized predicted values. Independence of residuals was assessed using the Durbin–Watson statistic. Multicollinearity among predictors within each model was evaluated using tolerance values, variance inflation factors (VIFs), and collinearity diagnostics. Influential observations were assessed using Cook’s distance, Mahalanobis distance, leverage values, and standardized residuals.

Regression results are presented as unstandardized regression coefficients (B), standardized regression coefficients (*β*), 95% confidence intervals (95% CI), and corresponding *p* values. Missing data were not imputed because missingness largely reflected non-completion of successive questionnaire sections, and the available data did not provide a sufficient basis for specifying and verifying an appropriate imputation model or missing-data mechanism. Descriptive analyses were therefore conducted on all available cases for each variable, with denominators reported separately, while the regression models were estimated on complete cases (*n* = 364). Statistical significance was set at alpha = 0.05.

## Results

### Sociodemographic characteristics of respondents

Of 608 GPs who accessed the survey, 61 discontinued before consent, leaving 547 eligible respondents. Completion then declined across sections: 471 completed the work-related items, and 416, 381, and 365 completed the knowledge, attitude, and practice scales respectively; complete-case regression models therefore drew on 364 respondents. Because completion varied across sections, analytic denominators ranged from 365 to 416 and are reported alongside each result. Women represented 82.8% of the sample (*n* = 453) and men 17.2% (*n* = 94), broadly consistent with the female-dominated composition of the Romanian GP workforce (approximately 78% women) (21). Most participants were over 50 years old (70.4%), followed by the 40–49 age group (21.4%), with only one participant younger than 30.

Education and professional experience: most respondents (58.7%) have one specialization; 11.3% have more than one specialization, 5.3% have a PhD and only 0.7% have a postdoctoral degree. 58.1% of respondents are primary physicians and 35.5% are specialist physicians and 6.4% have no specialized fellowship. Working experience: 76.8% of respondents have more than 15 years of working experience as general practitioners. Among those who reported the number of registered patients (*n* = 471), 29.9% had more than 2,500 registered patients, 27.0% had 1,500–2,000 patients, 23.8% had 2,000–2,500 patients, 18.1% had 800–1,500 patients, and 1.3% had fewer than 800 patients.

Regarding workload (*n* = 471), 4.7% of respondents saw fewer than 20 patients per day, 24.8% saw 21–24, 25.9% see between 25 and 29 patients per day, and 44.6% see more than 29 patients per day.

Among respondents reporting their number of MCI patients (*n* = 470), 45.5% had more than 51, 10% had 41–50 patients with MCI, only 8.1% of respondents have less than 10 patients diagnosed with MCI. 61.5% of respondents practice in the urban area.

Socio-demographic characteristics of respondents are presented in Table 2.

**Table 2:** Characteristics of general practitioners (GPs) involved in the study.

Variables	N of respondents	% of respondents
Age (years) (*N* = 547)		
<30	1	0.18
30–39	44	8.04
40–49	117	21.39
>50	385	70.38
Sex (*N* = 547)		
Female	453	82.8
Male	94	17.2
Marital status (*N* = 547)		
Married	407	74.41
Not married	58	10.60
Other	82	14.99
Working department (*N* = 547)		
Employed in a private practice	54	9.87
Employed in a Private medical clinic chain	12	2.19
Own medical practice	439	80.26
Other	42	7.68
Practice location (*N* = 540)		
Rural	208	38.5
Urban	332	61.5
Education (*N* = 547)		
Graduate	35	6.40
Postgraduate qualifications	96	17.55
One Specialization	321	58.68
More Specializations	62	11.33
PhD	29	5.30
Post Doc	4	0.73
Professional title (*N* = 547)		
Primary (attending) Physician	318	58.1
Specialist Physician	194	35.5
No specialized fellowship	35	6.4
Length of GP experience in years (*N* = 547)		
<5	46	8.41
5–9	41	7.50
10–14	40	7.31
>15	420	76.78
Daily patients (*N* = 471)		
<20	22	4.67
21–24	117	24.84
25–29	122	25.90
>29	210	44.59
Registered patients (*N* = 471)		
<800	6	1.27
800–1,500	85	18.05
1,500–2,000	127	26.96
2,000–2,500	112	23.78
>2,500	141	29.94
Have you attended MCI training? (*N* = 471)		
Yes	51	10.83
No	408	86.62
Do not know	12	2.55
Is MCI training useful for a GP (*N* = 471)		
Yes	371	78.77
No	36	7.64
Do not know	64	13.59
How many of your patients are diagnosed with MCI? (*N* = 470)		
1–10	38	8.1
11–20	51	10.9
21–30	61	13.0
31–40	59	12.6
41–50	47	10.0
>51	214	45.5

*N* = number of respondents.

### Knowledge, attitudes, and practice of GPs toward MCI detection and management

On average, the respondents obtained an MCI knowledge score of 33.20 (SD = 16.37). No respondent achieved a full knowledge score, only two respondents provided 7 correct answers while 12 respondents provided no correct answer. Relatively higher levels of knowledge were reflected in the questions associated with the specialists involved in diagnosis and management of MCI and nonpharmacological interventions efficient for MCI management, with more than 50% of respondents providing correct answers. The lowest levels of knowledge were observed for Petersen diagnostic criteria, with 6.5% correct responses, treatment of MCI, with 9.9% correct responses, and the most used MCI screening scale, with 10.8% correct responses. (Table 3). Knowledge levels: Most respondents (47.1%) have a low level of knowledge (between 11–25% correct), 41.1% of respondents have a moderate level of knowledge (26–50% correct). Very few respondents have a good level of knowledge (50–75% correct, 8.9% of respondents) and very low levels (1–10% correct 2.9% of respondents). No respondent had a very good level of knowledge. Importantly, only 416 completed the knowledge section of the survey.

**Table 3:** Knowledge, attitudes and practices regarding MCI detection and management.

Knowledge item	Incorrect (%)	Correct (%)
K1. What is the estimated percentage of people over 60 years old who have MCI in community setting?	62	38
K2. Which are modifiable risk factors affecting cognitive function?	79.3	20.7
K3. Which of the following is required to diagnose MCI according to Petersen diagnosis criteria?	93.5	6.5
K4. Which of the following doctors are necessary in MCI diagnosis?	15.1	84.9
K5. Which one of the following is the most used MCI screening scale?	89.2	10.8
K6. Which statement is true concerning treatment of MCI?	90.1	9.9
K7. What is the average progression rate after onset of MCI?	71.9	28.1
K8. What are effective non-pharmacological MCI interventions?	33.2	66.8

Attitude item	Strongly disagree (%)	Disagree (%)	Unsure (%)	Agree (%)	Strongly agree (%)
A1. MCI is not a disease, but a normal aging process.	25.2	36.7	10.5	23.6	3.9
A2. There are more advantages than disadvantages to finding out if someone has MCI.	6	5	6.6	47	35.4
A3. All patients suspected of MCI should undergo a diagnostic evaluation	1	0.5	1	57.5	39.9
A4. Early recognition and management can delay the progression to Alzheimer’s disease.	0.8	1	6.8	52.8	38.6
A5. There are more advantages than disadvantages to manage MCI patients with medicine	1.6	7.1	25.5	47	18.9
A6. There are more advantages than disadvantages to manage MCI patients with non-pharmaceutical methods.	1.3	3.9	24.7	48.8	21.3
A7. Patients with dementia can be a drain on medical and social resources	2.9	8.4	7.6	43.3	37.8
A8. Disclosure of disease could cause stress and frustration to patients and their families.	5.2	17.1	10.8	51.7	15.2
A9. Disclosure of disease could cause embarrassment or discomfort for doctors.	19.2	43.6	11.3	21.3	4.7
A10. Being diagnosed with MCI could provide some hope for patients compared with being diagnosed with Alzheimer’s disease.	0.8	0.5	5.5	66.7	26.5
A11. GPs should be involved in MCI detection, diagnosis and management	0.5	0.5	1	52	45.9

Practice items	Never %	Seldom %	Sometimes %	Usually %	Always %
P1. I take memory disorder as the criteria for MCI detection	0.8	8.2	34.0	34.2	22.7
P2. I take psychiatric symptoms as the criteria for MCI detection	6.6	35.6	27.4	23.0	7.4
P3. I would ask if a patient had Alzheimer’s disease family history	3.3	15.1	16.2	29.0	36.4
P4. I think that it is important to detect MCI risk factors	1.1	2.7	16.4	26.0	53.7
P5. I use the MCI screening methods during patient visits	15.3	36.2	19.5	17.8	11.2
P6. I would consult with specialists for final diagnosis by transference	3.6	10.7	19.5	23.0	43.3
P7. I would discuss the probable diagnosis with the patient.	2.5	11.5	24.7	35.1	26.3
P8. I would discuss the probable diagnosis with the family.	1.6	11.5	20.8	34.8	31.2
P9. I would prescribe medications recommended by specialists	1.9	4.1	15.9	32.6	45.5
P10. I would provide non-pharmacological interventions.	2.5	8.2	20.3	26.6	42.5
P11. I consider that frailty and MCI are linked	12.6	18.6	26.0	26.6	16.2

Mean practice score was 73.01 (SD = 11.53). Considering participants who responded “always” or “usually,” less than half of respondents (*n* = 365) used MCI screening methods during patient visits (29.0%). Similarly, less than half of respondents consider that frailty and MCI are linked (42.8%) or consider psychiatric symptoms as the criteria for MCI detection (30.4%). More than half of the respondents would consider memory disorder as the criteria for MCI detection (57%). Over 60% of respondents would always/usually discuss the probable diagnosis with the patient (61.4%) would ask if a patient has Alzheimer’s disease family history (65.4%) would discuss the probable diagnosis with the family (66%), consult with specialists for final diagnosis (66.3%) and provide non-pharmacological interventions (69.1%).

Mean attitude score was 73.26 (SD = 6.96). Most respondents would consider MCI as a disease condition (61.9%) while 27.5% consider MCI as a normal aging process. The vast majority of respondents consider that there are more advantages than disadvantages to finding out if someone has MCI (82.4%), that all patients suspected of MCI should undergo a diagnostic evaluation (97.4%) and that early recognition and management can delay the progression to Alzheimer’s disease (91.4%) and that GPs should be involved in MCI detection, diagnosis and management (97.9%). Overall, 65.9% of respondents endorsed the benefits of pharmacological treatment, while 70.1% indicated that non-pharmacological approaches are effective in managing MCI. Most respondents are concerned that patients with dementia can be a drain on medical and social resources (81.1%) and that disclosure of disease could cause stress and frustration to patients and their families (66.9%), however, only 26% of respondents are concerned that disclosure of disease could cause embarrassment or discomfort for doctors. Descriptive statistics for knowledge, attitude, and practice scores are presented in Table 4. The frequencies of responses for each item across the three scales are reported in Table 3.

**Table 4:** Descriptive statistics for knowledge, attitudes and practice scores.

Variable	*N*	Mean	SD	Minimum	Maximum
Knowledge score	416	33.20	16.37	0.00	87.50
Attitudes score	381	73.26	6.96	36.36	92.73
Practices score	365	73.01	11.53	20	100

*N* = number of respondents; *M* = mean; SD = standard deviation.

The distributional properties of the Practice, Knowledge, and Attitudes scores were assessed using skewness, kurtosis, and the Shapiro–Wilk test. The Practice score demonstrated moderate negative skewness and elevated kurtosis, indicating a left-skewed and leptokurtic distribution. The Knowledge score showed slight positive skewness and approximately normal kurtosis, suggesting a relatively symmetric distribution. The Attitudes score was approximately symmetric but exhibited moderate kurtosis. Results of the Shapiro–Wilk test indicated that all variables significantly deviated from normality (*p* < 0.001). Overall, although some skewness and kurtosis values were within acceptable ranges, the normality assumption was not met for any of the variables (Table 1).

### Factors associated with knowledge, attitudes, and practice

#### Correlational analyses

Spearman’s rank-order correlations were conducted to examine the relationships among practice, knowledge, and attitude scores and the sociodemographic and professional experience variables. The results indicated statistically significant, albeit weak, positive associations between practice scores and the number of daily patients, *r*ₛ(365) = 0.11, *p* = 0.039; knowledge scores, *r*ₛ(365) = 0.18, *p* = 0.001; and attitude scores, *r*ₛ(365) = 0.23, *p* < 0.001. These findings suggest that higher knowledge and more positive attitudes are modestly associated with better practice scores.

Additionally, a weak positive correlation was observed between attitude scores and the number of daily patients, *r*ₛ(381) = 0.11, *p* = 0.033 and the number of MCI patients registered with a GP, *r*ₛ(380) = 0.134, *p* = 0.009, indicating that GPs with higher patient volumes and more experience with MCI patients tended to report slightly more positive attitudes.

No other statistically significant correlations were identified among the variables of interest.

#### Group differences by MCI training

Mann–Whitney U tests were conducted to examine differences in knowledge, attitude, and practice scores between participants who had attended MCI training and those who had not. All statistical tests were conducted using nonparametric methods due to the distributional characteristics of the data.

Participants who had attended MCI training scored higher on both practice (mean rank = 215.62 vs. 172.95; *U* = 4993.00, *z* = −2.53, *p* = 0.011, r = 0.13) and knowledge (mean rank = 231.10 vs. 199.89; *U* = 7139.00, *z* = −1.77, *p* = 0.077, r = 0.09), though the knowledge difference did not reach significance. Attitude scores, by contrast, were lower among trained participants (mean rank = 156.17 vs. 190.01; *U* = 5881.50, *z* = −1.97, *p* = 0.049, *r* = 0.10). This negative training–attitude association was subsequently reproduced in the adjusted regression model (B = −2.609, *p* = 0.009), indicating it was not an artifact of the modelling approach.

#### Gender and age-group differences

No statistically significant differences were found between male and female participants in knowledge scores, *U* = 11270.00, *z* = −0.94, *p* = 0.346, r = 0.05; attitude scores, *U* = 10327.00, *z* = −0.38, *p* = 0.701, r = 0.02; or practice scores, *U* = 9501.00, z = −0.46, *p* = 0.643, *r* = 0.03. All observed effects were small in magnitude.

Similarly, no statistically significant differences were observed between the four age-group levels in knowledge scores, H(2) = 1.09, *p* = 0.581, η^2^ = 0.003; attitude scores, H(2) = 4.39, *p* = 0.111, η^2^ = 0.012; or practice scores, H(2) = 3.46, *p* = 0.177, η^2^ = 0.010. These effect sizes indicate negligible to small effects, suggesting that age group was not meaningfully associated with differences in knowledge, attitudes, or practice.

#### Differences by professional experience factors

A Kruskal–Wallis test indicated that there were no statistically significant differences across the four levels of length of professional experience in practice scores, H(3) = 3.76, *p* = 0.152; knowledge scores, H(3) = 0.33, *p* = 0.96; or attitude scores, H(3) = 1.88, *p* = 0.60; or across the four levels of the number of daily patients in practice scores, H(3) = 6.35, *p* = 0.096; knowledge scores, H(3) = 4.91, *p* = 0.178; or attitude scores, H(3) = 4.58, *p* = 0.205.

There were no statistically significant differences between attending physicians, specialist physicians and respondents with no specialized fellowship in practice scores, H(2) = 4.53, *p* = 0.104; knowledge scores, H(2) = 1.93, *p* = 0.38; or attitude scores, H(2) = 1.62, *p* = 0.45. Similarly, no statistically significant differences were observed between general practitioners practicing in rural and urban settings in practice scores, *U* = 13,955.00, *z* = 1.42, *p* = 0.155; knowledge scores, *U* = 18,665.00, *z* = −1.16, *p* = 0.246; or attitude scores, *U* = 15,582.00, *z* = −1.22, *p* = 0.223.

### Multiple linear regression analyses

Three multiple linear regression analyses were conducted to examine predictors of practice, knowledge, and attitudes.

Prior to conducting the regression analyses, the assumptions of multiple linear regression were evaluated for all three models. Visual inspection of histograms, normal probability (P–P) plots, and standardized residual scatterplots indicated no substantial deviations from normality, linearity, or homoscedasticity. Multicollinearity was not evident, with tolerance values ranging from 0.47 to 0.96 and variance inflation factors (VIFs) ranging from 1.04 to 2.12 across all models. Durbin–Watson statistics ranged from 1.90 to 2.05, indicating independence of residuals. Furthermore, Cook’s distance values were low in all models (maximum = 0.098), suggesting the absence of influential observations.

Factors associated with knowledge regarding MCI.

A multiple linear regression analysis was performed to identify factors independently associated with family physicians’ knowledge regarding MCI. The overall model was statistically significant, *F*(9, 354) = 2.54, *p* = 0.008, explaining 6.1% of the variance in knowledge scores (*R*^2^ = 0.061, adjusted *R*^2^ = 0.037).

Higher practice scores [*B* = 0.192, 95% CI (0.042, 0.343), *β* = 0.136, *p* = 0.012] and higher educational attainment [*B* = 1.434, 95% CI (0.32, 2.547), *β* = 0.140, *p* = 0.012] were independently associated with higher knowledge scores. Attitude scores, professional title, years of GP experience, attendance at MCI-related training, number of registered patients, number of daily patients and number of patients with MCI were not significantly associated with knowledge after adjustment for the other predictors (Table 5).

**Table 5:** Factors associated with MCI knowledge, attitudes and practices among general practitioners.

Predictor	Knowledge B (95% CI)	*β*	*p*	Attitudes B (95% CI)	*β*	*p*	Practices B (95% CI)	*β*	*p*
Knowledge score	—	—	—	0.034 (−0.010, 0.078)	0.079	0.130	**0.091 (0.020, 0.163)**	**0.130**	**0.012**
Attitudes score	0.190 (−0.056, 0.437)	0.082	0.130	—	—	—	**0.334 (0.167, 0.500)**	**0.204**	**< 0.001**
Practices score	**0.192 (0.042, 0.343)**	**0.136**	**0.012**	**0.126 (0.063, 0.189)**	**0.206**	**< 0.001**	—	—	—
Education	**1.434 (0.320, 2.547)**	**0.140**	**0.012**	−0.164 (−0.639, 0.311)	−0.037	0.498	−0.605 (−1.376, 0.166)	−0.084	0.124
Professional title	0.786 (−2.290, 3.862)	0.028	0.616	0.025 (−1.277, 1.326)	0.002	0.970	0.843 (−1.275, 2.960)	0.043	0.434
Years of GP experience	0.199 (−1.694, 2.092)	0.011	0.836	0.256 (−0.544, 1.056)	0.033	0.530	−0.336 (−1.640, 0.967)	−0.027	0.612
Daily patients	−1.896 (−4.339, 0.547)	−0.108	0.128	0.488 (−0.547, 1.523)	0.065	0.355	**2.557 (0.890, 4.223)**	**0.207**	**0.003**
Registered patients	0.773 (−1.231, 2.778)	0.053	0.449	0.310 (−0.538, 1.157)	0.049	0.473	**−1.713 (−3.082, −0.343)**	**−0.167**	**0.014**
Attended MCI training	3.019 (−1.613, 7.650)	0.068	0.201	**−2.609 (−4.552, −0.665)**	**−0.136**	**0.009**	**3.660 (0.486, 6.834)**	**0.117**	**0.024**
Number of patients with MCI	−0.209 (−1.151, 0.734)	−0.023	0.663	**0.397 (0.001, 0.794)**	**0.102**	**0.049**	−0.164 (−0.813, 0.485)	−0.026	0.619

*N* = 364 complete cases. Unstandardized regression coefficients (B), 95% confidence intervals (CIs) for B, standardized regression coefficients (*β*), and p values are presented. MCI training was entered as a binary predictor [0 = no or do not know (reference), 1 = yes]. Ordered multi-category predictors were entered as ordinal variables using increasing category codes; their coefficients therefore represent a one-category increase. Detailed coding is provided in the Methods. Statistically significant associations (*p* < 0.05) are shown in bold. CI = confidence interval; GP = general practitioner; MCI = mild cognitive impairment.

Factors associated with attitudes toward MCI.

A second multiple linear regression model examined factors independently associated with physicians’ attitudes toward MCI. The model was statistically significant, *F*(9, 354) = 4.23, *p* < 0.001, explaining 9.7% of the variance in attitude scores (*R*^2^ = 0.097, adjusted *R*^2^ = 0.074).

Higher practice scores were independently associated with more favorable attitudes [*B* = 0.126, 95% CI (0.063, 0.189), *β* = 0.206, *p* < 0.001]. Physicians caring for a larger number of patients with MCI also reported more favorable attitudes [*B* = 0.397, 95% CI (0.001, 0.794), *β* = 0.102, *p* = 0.049]. Attendance at MCI-related training courses was independently associated with lower attitude scores [*B* = −2.609, 95% CI (−4.552, −0.665), *β* = −0.136, *p* = 0.009]. Knowledge scores, education, professional title, years of GP experience, and number of registered patients and number of daily patients were not independently associated with attitudes (Table 5).

Factors associated with practices regarding patients with MCI.

A third multiple linear regression analysis was conducted to identify factors associated with physicians’ practices regarding patients with MCI. The model was statistically significant, *F*(9, 354) = 4.51, *p* < 0.001, explaining 10.3% of the variance in practice scores (*R*^2^ = 0.103, adjusted *R*^2^ = 0.080).

Higher knowledge scores [*B* = 0.091, 95% CI (0.020, 0.163), *β* = 0.130, *p* = 0.012], more favorable attitudes [*B* = 0.334, 95% CI (0.167, 0.500), *β* = 0.204, *p* < 0.001], higher daily patient volume [*B* = 2.557, 95% CI (0.890, 4.223), *β* = 0.207, *p* = 0.003], and attendance at MCI-related training [*B* = 3.660, 95% CI (0.486, 6.834), *β* = 0.117, *p* = 0.024] were independently associated with better practice scores. Conversely, physicians with larger registered patient lists had lower practice scores [*B* = −1.713, 95% CI (−3.082, −0.343), *β* = −0.167, *p* = 0.014]. Education, professional title, years of experience, and the number of patients with MCI were not significant predictors (Table 5). Model fit statistics for the three regression models are summarised in Table 6.

**Table 6:** Model fit statistics for the three multiple linear regression models.

Outcome	*R* ^2^	Adjusted *R*^2^	*F*(df1, df2)	*p*
Knowledge	0.061	0.037	2.54 (9,354)	0.008
Attitudes	0.097	0.074	4.23 (9,354)	<0.001
Practices	0.103	0.080	4.51 (9,354)	<0.001

*R*^2^ = coefficient of determination; F(df1, df2) = F-statistic for overall model fit; *N* = 364 complete cases for all three models.

## Discussion

### Principal findings

To our knowledge, this is the first national study to examine Romanian GPs’ knowledge, attitudes and reported practices regarding MCI detection and management. The findings identify a clear implementation gap between support for early detection and its translation into routine primary care. Although 97.9% of GPs agreed that early detection of MCI should form part of primary care, only 29.0% reported routinely using cognitive screening tools. This gap was accompanied by limited MCI-specific training, with 86.6% reporting no previous training, while 78.8% considered MCI training useful for GPs.

The regression models did not identify a common set of predictors across all three KAP domains. Instead, knowledge, attitudes and practices showed partly different patterns of association, indicating that primary care preparedness for MCI is multidimensional rather than reducible to a single underlying construct. In the practice model, more favorable attitudes were independently associated with higher reported practice scores (*B* = 0.334, *β* = 0.204, *p* < 0.001), as were greater knowledge (*B* = 0.091, *β* = 0.130, *p* = 0.012), previous attendance at MCI-specific training (*B* = 3.660, *β* = 0.117, *p* = 0.024) and higher daily patient volume. Conversely, larger registered patient lists were associated with lower reported practice scores. These findings suggest that implementation depends not simply on what GPs know, but also on how they perceive MCI care, their exposure to relevant training and the organizational context in which they work.

This implementation gap is particularly relevant in Romania, where a high estimated prevalence of MCI among older adults (1) coincides with population aging (2) and the wider health and societal burden associated with cognitive impairment and dementia (3). In the absence of an organised national programme for systematic early detection, opportunities to identify early cognitive decline are likely to depend substantially on routine primary care encounters. Strengthening this role therefore requires more than increasing awareness: it requires the professional capability, practical pathways and organizational capacity to translate early detection into routine clinical practice.

### Knowledge and preparedness for early detection

Knowledge represented an important but incomplete component of preparedness. The most prominent gaps concerned areas directly relevant to early identification, including diagnostic criteria, cognitive screening instruments and modifiable risk factors. These are clinically important because regular contact with older patients creates opportunities for early recognition only if clinicians can identify meaningful cognitive change, select appropriate assessment approaches and determine appropriate next steps.

These findings are consistent with international evidence. Chodosh et al. found that physicians recognized MCI less reliably than established dementia (22), while Kaduszkiewicz et al. reported low detection of MCI by GPs in Germany (23). A meta-analysis of eight studies similarly found that GPs recognized fewer than half of individuals with MCI by contemporaneous clinical judgement (24). More recent KAP studies have continued to identify limited familiarity with MCI, diagnostic uncertainty and inconsistent screening and management (17, 18, 25). The persistence of these findings across health systems suggests that difficulties in early detection reflect not only individual knowledge gaps, but also the extent to which MCI assessment has been incorporated into routine primary care.

The adjusted analyses add an important dimension to this interpretation. Knowledge was independently associated with reported practice (*B* = 0.091, *β* = 0.130, *p* = 0.012), and higher educational attainment was independently associated with greater knowledge (*B* = 1.434, *β* = 0.140, *p* = 0.012). Notably, previous MCI-specific training was not independently associated with knowledge after adjustment. This suggests that exposure to training, as currently delivered, may not consolidate the technical knowledge required for early detection. However, knowledge was only one of several factors associated with practice, indicating that greater knowledge alone is unlikely to be sufficient to close the implementation gap.

This distinction has practical implications. Educational interventions may strengthen clinicians’ understanding of MCI, but translation into routine practice is also likely to depend on attitudes toward early detection, practical competence in assessment and the conditions under which primary care is delivered.

### From favorable attitudes to clinical practice

GPs generally expressed favorable attitudes toward involvement in MCI detection and management. The finding that 97.9% supported early detection as part of primary care suggests that limited implementation is unlikely to reflect resistance to the principle of early identification. This is consistent with the European MOPEAD survey, in which support for early diagnosis coexisted with substantial barriers to implementation (19).

At the same time, favorable attitudes coexisted with uncertainty about whether MCI represents normal aging and concerns about the consequences of diagnostic disclosure. This combination highlights an important distinction between supporting early detection in principle and feeling sufficiently prepared and supported to implement it consistently.

The multivariable findings reinforce the importance of this distinction. Attitudes were independently associated with reported practices (*B* = 0.334, *β* = 0.204, *p* < 0.001), even after adjustment for other factors in the model. This suggests that how GPs conceptualise the value and feasibility of MCI detection may be particularly relevant to whether MCI-related knowledge is translated into clinical action.

However, the relationship between knowledge, attitudes and practice does not appear to follow a simple linear pathway. Knowledge, attitudes and practices showed different patterns of association in the adjusted models, while reported practice was independently related to several domains. Primary care readiness for MCI may therefore be better understood as the product of multiple interacting dimensions, rather than a straightforward progression from knowledge to favorable attitudes and then to practice.

The descriptive pattern of reported clinical activities supports this interpretation. GPs were more likely to report actions undertaken once cognitive impairment was already suspected, such as discussing probable MCI, obtaining family history, consulting specialists and recommending non-pharmacological interventions, than to report routine use of cognitive screening tools, which was reported by only 29.0%.

This may indicate greater preparedness for reactive management than proactive case identification. The distinction is important: responding appropriately once cognitive concerns become apparent is not equivalent to having a systematic mechanism for recognising cognitive decline earlier in its course. This disconnect between willingness, knowledge and systematic detection lies at the centre of the implementation gap identified in this study.

### MCI-specific training: an important but complex factor

Training emerged as a particularly informative factor because its associations differed across KAP domains. Only 10.8% of respondents had received MCI-specific training, while 78.8% reported a need for further education, indicating a substantial mismatch between training exposure and perceived need.

In the adjusted analyses, previous MCI-specific training was associated with higher reported practice scores (*B* = 3.660, *β* = 0.117, *p* = 0.024), but not with knowledge scores after adjustment. This suggests that training may encourage engagement with MCI-related clinical activities without necessarily producing measurable gains in technical knowledge.

Unexpectedly, previous MCI-specific training was inversely associated with attitude scores [*B* = −2.609, 95% CI (−4.552, −0.665), *β* = −0.136, *p* = 0.009]. This finding should be interpreted cautiously, given the cross-sectional design and the relatively small number of physicians with MCI-specific training. Several explanations are possible, including differences in clinical exposure, self-selection into training, or greater awareness of the complexities involved in MCI assessment and management among trained physicians, although the present data cannot distinguish between these explanations. Notably, this finding coexists with a positive association between training and reported practice, suggesting that the relationship between training and the different KAP domains may be more complex. Further research is needed to clarify the mechanisms underlying this association.

The three KAP domains should therefore not be treated as interchangeable measures of a single construct. An intervention targeting one domain may not produce corresponding changes in the other two. Accordingly, training strategies should extend beyond information to include practical assessment skills, interpretation of brief cognitive assessment tools, communication of diagnostic uncertainty, risk-factor counselling and appropriate use of referral pathways (17, 18, 25, 26).

### Workload and organizational capacity

The adjusted analyses also indicate that reported MCI-related practices are shaped by the context in which GPs work. Workload-related factors remained independently associated with practice after adjustment, but the direction of association differed according to the workload measure.

Higher daily patient volume was associated with higher reported practice scores, whereas larger registered patient lists were associated with lower practice scores. Although these findings require cautious interpretation, they suggest that workload is not a unidimensional construct.

Greater daily patient exposure may increase opportunities to encounter cognitive concerns and accumulate relevant clinical experience. In contrast, responsibility for a larger registered population may reflect cumulative clinical, continuity-of-care and administrative demands that constrain the time available for structured cognitive assessment. These explanations remain hypothesis-generating, but the contrasting associations suggest that clinical exposure and organizational burden may have different relationships with MCI-related practice.

This distinction helps contextualise the implementation gap. High patient exposure may increase opportunities to recognize MCI, while competing workload demands may simultaneously limit the capacity to undertake systematic assessment. Even knowledgeable and motivated GPs may struggle to incorporate cognitive screening and follow-up into routine practice when consultation time, workflow or referral capacity are constrained.

This interpretation is consistent with previous research identifying time pressure, workload and organizational barriers as important obstacles to cognitive assessment in primary care (26). It also reinforces a central implication of the present study: educational interventions alone are unlikely to be sufficient if the clinical environment does not support implementation.

### A multidimensional model of primary care readiness for MCI

The regression results indicate that knowledge, attitudes and practices show partly distinct patterns of association. This is one of the more important analytical findings of the study because it challenges the assumption that MCI preparedness can be represented by a single continuum.

The models explained different proportions of variance in each domain, with adjusted R^2^ values of 0.037 for knowledge, 0.074 for attitudes and 0.080 for practices. Explanatory power was comparable for attitudes and practices and lowest for knowledge, and substantial unexplained variation remained across all three outcomes.

The modest explanatory power of the models should not obscure the broader pattern of associations. Rather, it indicates that preparedness is likely to reflect a wider set of influences than those measured here. Knowledge appears partly related to educational factors; attitudes show a different pattern of associations; and reported practices appear to reflect the combined influence of knowledge, attitudes, training and practice context.

Conceptually, these findings suggest that readiness for early MCI detection involves at least three related components: capability, including relevant knowledge and skills; professional orientation, including beliefs about the value and feasibility of early detection; and implementation capacity, including training exposure, workload and the organizational conditions required to act.

This framework may help explain why favorable attitudes toward early detection coexist with limited routine screening. A GP may support early identification and possess relevant knowledge but still face time constraints, lack practical assessment tools or have limited access to specialist services required for appropriate follow-up. Conversely, greater clinical exposure may be associated with more frequent MCI-related activity even where formal knowledge or training is incomplete.

### Public health and implementation implications

The findings have important implications for public health planning in Romania. Applying available prevalence estimates to Romania’s older population suggests that approximately one million older adults may be living with MCI (1, 27). Although necessarily approximate, this estimate illustrates the potential scale of need and reinforces that early cognitive decline is a population-level clinical issue.

MCI may represent an important window for secondary prevention and risk reduction. Timely recognition can enable investigation of potentially reversible contributors, management of modifiable risk factors, monitoring of cognitive trajectories and appropriate follow-up before substantial functional decline develops (7, 14, 15). Realising this potential, however, requires closing the gap between professional willingness and implementation capacity.

### The present findings suggest three priorities

First, targeted workforce development is needed. Only 10.8% of respondents had received MCI-specific training, while 78.8% reported a need for further education, indicating a substantial unmet demand. The adjusted association between training and reported practice supports its potential relevance. Training should, however, extend beyond factual knowledge to include practical use and interpretation of brief cognitive assessment tools, communication of uncertainty, risk-factor counselling and referral pathways.

Second, GPs require clear and feasible guidance for early cognitive assessment. The contrast between strong support for early detection (97.9%) and low routine use of screening tools (29.0%) suggests that willingness alone does not translate into systematic assessment. Guidance should clarify whom to assess, under what circumstances, which validated tools are feasible in primary care, how results should be interpreted and what should follow an abnormal assessment (13–15). Such recommendations must be realistic within the time and workload constraints of Romanian general practice.

Third, organizational capacity must form part of implementation planning. The independent associations between workload characteristics and reported practice indicate that structural conditions may facilitate or constrain MCI-related activity. Training clinicians without addressing workflow, consultation constraints and access to appropriate referral pathways may therefore have limited impact.

Overall, the findings argue against a single-component response. Strengthening primary care preparedness for MCI is likely to require a coordinated approach combining professional capability, practical implementation guidance and organizational support.

These implications may extend beyond Romania. Many aging health systems face increasing demand for earlier recognition of cognitive decline alongside substantial pressure on primary care. The consistency of the present findings with international evidence (17–19, 22–26) suggests that the gap between support for early detection and the capacity to implement it may represent a broader challenge for health systems responding to population aging.

### Strengths and limitations

This study has several strengths. To our knowledge, it is the first national assessment of Romanian GPs’ knowledge, attitudes and reported practices regarding MCI detection and management. It included a large sample of GPs from different regions and both urban and rural settings. Although sampling was non-probabilistic, the gender and urban–rural composition broadly approximated the Romanian GP workforce, providing some reassurance regarding sample coverage.

A further strength is the inclusion of multivariable analyses examining factors independently associated with each KAP domain. This allowed the study to move beyond descriptive estimates and identify distinct patterns of association across knowledge, attitudes and reported practices.

Several limitations should nevertheless be considered. Valid response numbers decreased across the knowledge, attitude and practice sections and were lower in some multivariable analyses, and completers were not compared with non-completers. Differential attrition may therefore have influenced both score estimates and model results.

The cross-sectional design precludes causal or directional interpretation of the observed associations. This is particularly important for findings such as the inverse association between MCI-specific training and attitudes, where reverse or selective exposure cannot be excluded. All measures were self-reported and may also be affected by recall and social desirability bias, particularly for practice-related behaviors.

Recruitment through open online channels meant that a conventional response rate could not be calculated and the representativeness of the sample could not be formally assessed. The convenience sample may therefore be affected by self-selection bias, potentially over-representing physicians with greater interest in cognitive health or MCI and limiting generalizability despite broad demographic coverage.

The adapted questionnaire was reviewed with practicing GPs for clarity, clinical relevance and contextual appropriateness but was not formally psychometrically validated in the Romanian context. Internal consistency and factor structure of the adapted scales were not established, and the knowledge categories were used descriptively rather than as validated competency thresholds. This consideration applies particularly to the practice scale: in adapting the instrument, several practice items were reworded from hypothetical intention (“I would…”) toward current reported behavior (e.g., “I use MCI screening methods during patient visits”) or toward the perceived importance of an activity (e.g., “I think it is important to detect MCI risk factors”). The resulting score therefore combines reported behavior, intention and perceived importance, and is best interpreted as a broad composite of self-reported practice orientation rather than a unidimensional behavioral measure. Accordingly, associations involving the practice score, including the regression findings, should be interpreted in relation to this broader composite construct. Its distribution was markedly left-skewed and leptokurtic (Table 1), consistent with a ceiling tendency; non-parametric methods were used for bivariate analyses, and regression estimates involving the practice score should be read with corresponding caution.

These adaptations were, however, mostly minor and item-specific, terminological adjustments, contextual clarifications, and the removal of items inapplicable to Romanian primary care, rather than a wholesale redevelopment of the instrument, which was itself adapted from a previously published and used questionnaire [Lu et al.]. Expert review by practicing GPs supported the face and content validity of the adapted items, and the core constructs were preserved. Formal psychometric validation nonetheless remains a necessary next step, and the present findings should be read as a first national estimate rather than a definitive measurement.

Accordingly, health-system preparedness is considered here from the perspective of primary care’s contribution to early MCI detection, not as a comprehensive assessment of the health system.

Finally, the multivariable models explained a modest proportion of variance across all three domains (adjusted *R*^2^ = 0.037 for knowledge, 0.074 for attitudes and 0.080 for practices), indicating that much of what shapes MCI-related behavior lies outside the variables measured in this study. Potentially relevant factors may therefore not have been captured, including consultation duration, previous geriatric or neurological experience, access to specialist assessment, reimbursement structures and organizational characteristics of individual practices. Future research should examine these factors using longitudinal and implementation-focused designs capable of testing which interventions lead to meaningful changes in clinical practice.

## Conclusion

This national study suggests that Romanian primary care has an important but insufficiently supported role in the early detection of MCI. GPs largely endorsed involvement in detecting and managing the condition, yet this favorable orientation coexisted with substantial knowledge gaps, limited routine use of screening tools and low participation in MCI-specific training. The capacity of primary care to contribute systematically to the detection of early cognitive decline at population level therefore remains constrained.

The findings further suggest that primary care preparedness for MCI is multidimensional, with knowledge, attitudes and practices associated with partly distinct factors. Reported practices were independently associated with attitudes, knowledge, previous MCI-specific training and workload-related factors, although the regression models explained only a modest proportion of variance. These findings should be interpreted as associations rather than causal effects, but they suggest that clinical implementation depends on more than individual knowledge or willingness. The differing associations of MCI-specific training across the KAP domains also support a broader approach to workforce education, extending beyond factual knowledge to include practical assessment skills, communication of diagnostic and prognostic uncertainty and appropriate use of existing referral pathways.

The principal public health concern is the mismatch between the need for early cognitive assessment and the current capacity of Romanian primary care to provide it systematically. In a country where close to one in five inhabitants is aged 65 years or over (27), where the estimated prevalence of MCI among older adults is among the highest in Europe (1), and where no organised national programme for systematic early detection exists, strengthening primary care preparedness should be a priority for public health planning.

No single intervention is likely to close this gap. Addressing it will require a coordinated approach combining practical professional training, feasible guidance for cognitive assessment in general practice and sufficient time for structured assessment. Longitudinal and intervention studies are needed to clarify the direction of the observed associations and determine which approaches lead to measurable improvements in early detection. These challenges are unlikely to be unique to Romania, as other aging health systems may face similar gaps between population need and the capacity to respond.

## Data Availability

The datasets presented in this study can be found in online repositories. The names of the repository/repositories and accession number(s) can be found at: Open Science Framework (OSF): https://osf.io/cd2jb/overview?view_only=ee707c281e4d41bf9d4fc892229b1bf4.
