# Supplementary material for: Health-system preparedness for early detection of mild cognitive impairment: primary-care evidence from a national study among general practitioners in Romania
Source: Front Public Health. 2026 Oct 23;14:1969120. doi: 10.3389/fpubh.2026.1969120 (PMC13739480; doi:10.3389/fpubh.2026.1969120)
Supplement: Supplementary file 1 [file Data_Sheet_1.PDF]

Copyright and licence notice
The original questionnaire reproduced in this supplementary file was published as Additional file 2 accompanying the article by Lu et al. (2022). It is licensed under the Creative Commons Attribution 4.0 International License (CC BY 4.0; https://creativecommons.org/licenses/by/4.0/). The questionnaire was adapted for use in the present study. The changes made to adapt it to the Romanian primary care context are described in the item-by-item rationale provided below.

Source: Lu Y, Liu C, Fawkes S, Wang Z, Yu D. Knowledge, attitudes, and practice of general practitioners toward community detection and management of mild cognitive impairment: a cross-sectional study in Shanghai, China. BMC Primary Care. 2022;23:114. doi:10.1186/s12875-022-01716-9.

ORIGINAL QUESTIONNAIRE
developed by Lu et al. 2022

Survey on the detection and management of mild cognitive impairment in community health services

Dear physicians:

Greeting! We are conducting a questionnaire survey on the preparedness of general practice in detecting and managing mild cognitive impairment (MCI) in Shanghai. You are invited to complete this 20-minute questionnaire. Your responses are of great values. We hope to learn your experience and perceptions, which will help us determine how general practitioners (GPs) can effectively detect and manage MCI in Shanghai. The survey is completely voluntary and anonymously. Your responses will only be used for academic research only.

If you are interested in the survey, please read the following information statement carefully. Thank you very much for your participation.

Please make sure whether you`d like to take this survey：

☐ **Yes**
☐ **No**

Participant characteristics

1. Gender

☐Male
☐Female
☐Others

2. Age

☐ < 30
☐ 30-39
☐ 40-49
☐ ≥ 50

3. Marital status

☐Married
☐Unmarried
☐Other

4. **Educational level**
   ☐ Under bachelor
   ☐ Bachelor
   ☐ Postgraduate

5. **Working department**
   ☐General practice
   ☐ Public health
   ☐ Health administration
   ☐ Others
6. **Length of GP experience**
   ☐ < 5
   ☐ 5-9
   ☐ 10-14
   ☐ ≥15

7. **Professional title**
   ☐ Primary
   ☐ Middle
   ☐ Associate senior
   ☐ Senior

8. **Monthly income after tax**
   ☐ < ¥8000
   ☐ ¥8000-11999
   ☐ ¥12000-14999
   ☐ ≥ ¥15000

MCI detection and management context experience

1. **Daily visiting patients**
   ☐ < 80
   ☐ 80-99
   ☐ 100-119
   ☐ ≥120

2. **MCI training experience**
   ☐ Yes
   ☐ No
   ☐ Unsure

3. **MCI detection qualification**
   ☐Yes
   ☐No
   ☐Unsure

4. Proportion of patients with memory disorder last month

☐ 0
☐ <10%
☐ 10-29%
☐ ≥ 30%
☐ Unsure

5. Proportion of patients with psychiatric symptoms last month

☐ 0
☐ <10%
☐ 10-29%
☐ ≥ 30%
☐ Unsure

6. MCI detection and management experience

☐ Yes
☐ No
☐ Unsure

Knowledge of GPs toward MCI detection and management

1. What is the estimated percentage of people over 60 years old who have MCI in community setting?

☐ <10%
☐ 10-29%
☐ ≥ 30%
☐ Unsure

2. **Which are modifiable risk factors affecting cognitive function?**

☐ lack of exercise
☐ hearing loss
☐ depression
☐ moderate drink
☐ Unsure

3. Which of the following is NOT required to diagnose MCI according to Peterson diagnosis criteria?

☐ Subjective memory loss
☐ Impaired activity of daily living
☐ Evidence of cognitive deterioration
☐ Not clinically demented
☐ Unsure

4. Which of the following doctors are necessary in MCI diagnosis?

☐ Psychologist

☐ Psychiatrist

☐ Neurologist

☐ All of the above

☐ Unsure

5. Which one of the following is the most commonly-used MCI screening scale?

☐ The Montreal Cognitive Assessment (MoCA)

☐ Mini-Mental State Examination (MMSE)

☐ Clinic Dementia Rating Score (CDR)

☐ Activities of Daily Living (ADL)

☐ Unsure

6. Which statement is false concerning treatment of MCI?

☐ Donepezil should be prescribed

☐ Management of MCI may reverse symptoms

☐ It is important to rule out and treat reversible risks

☐ The cognitive function may reverse automatically in some MCI cases

☐ Unsure

7. What is the average progression rate after onset of MCI?

☐ <10%

☐ 10-19%

☐ 20-39%

☐ ≥40%

☐ Unsure

8. What are effective non-pharmacological MCI interventions?

☐ Aerobic exercise

☐ Mediterranean diet

☐ Music

☐ Social activities

☐ Donepezil effective effect

Attitudes of GPs toward MCI detection and management

	Strongly disagree	Disagree	Unsure	Agree	Strongly Agree
1. MCI is not a disease, but a degenerative aging process.	☐	☐	☐	☐	☐
2. There are more advantages than disadvantages to finding out if someone has MCI.	☐	☐	☐	☐	☐
3. All patients suspected of MCI should undergo a diagnostic evaluation.	☐	☐	☐	☐	☐
4. Early recognition and management can delay the progression to Alzheimer`s disease.	☐	☐	☐	☐	☐
5. There are more advantages than disadvantages to manage MCI patients with medicine.	☐	☐	☐	☐	☐
6. There are more advantages than disadvantages to manage MCI patients with non-pharmaceutical methods.	☐	☐	☐	☐	☐
7. Patients with dementia can be a drain on medical and social resources	☐	☐	☐	☐	☐
8. Disclosure of disease could cause stress and frustration to patients and their families.	☐	☐	☐	☐	☐
9. Disclosure of disease could cause embarrassment or discomfort for doctors.	☐	☐	☐	☐	☐
10. Being diagnosed with MCI could provide some hope for patients compared with being diagnosed with Alzheimer's` disease.	☐	☐	☐	☐	☐
11. MCI detection and management provide no economic benefits.	☐	☐	☐	☐	☐
12. It`s GPs responsibility to recognise MCI in the primary care setting.	☐	☐	☐	☐	☐
13. It`s GPs responsibility to managing MCI in the primary care setting.	☐	☐	☐	☐	☐

Practice of GPs toward MCI detection and management

	Always	Usually'	Sometimes	Seldom	Never	Not Applicable
1. I take memory disorder as the criteria for MCI detection	☐	☐	☐	☐	☐	☐
2. I take psychiatric symptoms as the criteria for MCI detection	☐	☐	☐	☐	☐	☐
3. I would ask if a patient has Alzheimer's` disease family history	☐	☐	☐	☐	☐	☐
4. I would detect MCI risk factors	☐	☐	☐	☐	☐	☐
5. I would utilise the MCI screening methods	☐	☐	☐	☐	☐	☐
6. I would get specialist advice for final diagnosis by transference	☐	☐	☐	☐	☐	☐
7. I would discuss the probable diagnosis with the patient.	☐	☐	☐	☐	☐	☐
8. I would discuss the probable diagnosis with the family.	☐	☐	☐	☐	☐	☐
9. I would coordinate support services.	☐	☐	☐	☐	☐	☐
10. I would prescribe medications	☐	☐	☐	☐	☐	☐
11. I would provide non-pharmacological interventions.	☐	☐	☐	☐	☐	☐

Romanian Adaptation of the MCI Knowledge, Attitudes and Practices Questionnaire (Lu et al., 2022): English Translation

Survey on the detection and management of mild cognitive impairment in community health services

Dear physicians:

Greeting! We are conducting a questionnaire survey on the preparedness of general practice in detecting and managing mild cognitive impairment (MCI) in Romania. You are invited to complete this 20-minute questionnaire. Your responses are of great values. We hope to learn your experience and perceptions, which will help us determine how general practitioners (GPs) can effectively detect and manage MCI in Romania. The survey is completely voluntary and anonymously. Your responses will only be used for academic research only.

If you are interested in the survey, please read the following information statement carefully. Thank you very much for your participation.

Please make sure whether you`d like to take this survey：

☐ **Yes**
☐ **No**

Participant characteristics

1. What is the setting of your current professional practice?

☐Rural
☐Urban

2. Sex

☐Male
☐Female
☐Other

3. Age

☐ < 30
☐ 30-39
☐ 40-49
☐ > 50

4. Marital status

☐Married
☐ Not married
☐Other

5. Working department

☐ Employed in a private practice
☐ Employed in a Private medical clinic chain
☐ Own medical practice
☐ Others

6. **Educational level**
   ☐ Graduate
   ☐ Postgraduate qualifications
   ☐ One Specialization
   ☐ More Specializations
   ☐ PhD
   ☐ Post Doc

7. **Professional title**
   ☐ Primary (attending) Physician
   ☐ Specialist Physician
   ☐ No specialized fellowship

8. **Length of GP experience**
   ☐ < 5
   ☐ 5-9
   ☐ 10-14
   ☐ > 15

MCI detection and management context experience

1. **Daily visiting patients**
   ☐ < 20
   ☐ 21-24
   ☐ 25-28
   ☐ >29

2. **How many patients are registered on your patient list?**
   ☐ < 800
   ☐ 800-1500
   ☐ 1500-2000
   ☐ 2000-2500
   ☐ >2500

3. **Have you received any training in the detection of mild cognitive impairment (e.g. MoCA certification or MMSE training)?**
   ☐ Yes
   ☐ No
   ☐ Do not know

4. **Do you consider it necessary to acquire specific competencies in the detection of cognitive impairment?**
   ☐ Yes
   ☐ No
   ☐ Do not know

5. **How many patients registered on your patient list have mild cognitive impairment (MCI)?**
   ☐ 1 - 10
   ☐ 11 - 20
   ☐ 21 - 30
   ☐ 31 - 40
   ☐ 41- 50
   ☐ > 51

Knowledge of GPs toward MCI detection and management

1. **What is the estimated percentage of people over 60 years old who have MCI in community setting?**
   ☐ <10%
   ☐ 10-29%
   ☐ ≥ 30%
   ☐ Unsure

2. **Which are modifiable risk factors affecting cognitive function?**
   ☐ reduced engagement in cognitively stimulating activities (e.g. reading, crossword puzzles, Sudoku)
   ☐ physical inactivity
   ☐ hearing loss
   ☐ depression
   ☐ moderate drink
   ☐ Unsure

3. **Which of the following is required to diagnose MCI according to Petersen diagnosis criteria?**
   ☐ Subjective memory loss
   ☐ Impaired activity of daily living
   ☐ Evidence of cognitive deterioration
   ☐ Not clinically demented
   ☐ Unsure

4. **Which of the following doctors are necessary in MCI diagnosis?**
   ☐ General practitioner
   ☐ Psychiatrist
   ☐ Neurologist
   ☐ Geriatrician
   ☐ All of the above
   ☐ Unsure

5. **Which one of the following is the most used MCI screening scale?**
   ☐ The Montreal Cognitive Assessment (MoCA)
   ☐ Mini-Mental State Examination (MMSE)
   ☐ Clinic Dementia Rating Score (CDR)
   ☐ Activities of Daily Living (ADL)
   ☐ Unsure

6. Which statement is true concerning treatment of MCI?

☐ Management of MCI may reverse symptoms

☐ It is important to rule out and treat reversible risks

☐ The cognitive function may reverse automatically in some MCI cases

☐ Unsure

7. What is the average progression rate after onset of MCI?

☐ <10%

☐ 10-19%

☐ 20-39%

☐ ≥40%

☐ Unsure

8. What are effective non-pharmacological MCI interventions?

☐ Aerobic exercise

☐ Mediterranean diet

☐ Music

☐ Social activities

☐ Cognitively stimulating activities (e.g. reading, crossword puzzles, Sudoku)

☐ All of the above

Attitudes of GPs toward MCI detection and management

	Strongly disagree	Disagree	Unsure	Agree	Strongly Agree
1. MCI is not a disease, but a normal aging process.	☐	☐	☐	☐	☐
2. There are more advantages than disadvantages to finding out if someone has MCI.	☐	☐	☐	☐	☐
3. All patients suspected of MCI should undergo a diagnostic evaluation	☐	☐	☐	☐	☐
4. Early recognition and management can delay the progression to Alzheimer's disease.	☐	☐	☐	☐	☐
5. There are more advantages than disadvantages to manage MCI patients with medicine	☐	☐	☐	☐	☐
6. There are more advantages than disadvantages to manage MCI patients with non-pharmaceutical methods.	☐	☐	☐	☐	☐
7. Patients with dementia can be a drain on medical and social resources	☐	☐	☐	☐	☐
8. Disclosure of disease could cause stress and frustration to patients and their families.	☐	☐	☐	☐	☐
9. Disclosure of disease could cause embarrassment or discomfort for doctors.	☐	☐	☐	☐	☐
10. Being diagnosed with MCI could provide some hope for patients compared with being diagnosed with Alzheimer's disease.	☐	☐	☐	☐	☐
11. GPs should be involved in MCI detection, diagnosis and management	☐	☐	☐	☐	☐

Practice of GPs toward MCI detection and management

	Always	Usually'	Sometimes	Seldom	Never	Not Applicable
1. I take memory disorder as the criteria for MCI detection	☐	☐	☐	☐	☐	☐
2. I take psychiatric symptoms as the criteria for MCI detection	☐	☐	☐	☐	☐	☐
3. I would ask if a patient had Alzheimer's disease family history	☐	☐	☐	☐	☐	☐
4. I think that it is important to detect MCI risk factors	☐	☐	☐	☐	☐	☐
5. I use the MCI screening methods during patient visits	☐	☐	☐	☐	☐	☐
6. I would consult with specialists for final diagnosis by transference	☐	☐	☐	☐	☐	☐
7. I would discuss the probable diagnosis with the patient.	☐	☐	☐	☐	☐	☐
8. I would discuss the probable diagnosis with the family.	☐	☐	☐	☐	☐	☐
9. I would prescribe medications recommended by specialists	☐	☐	☐	☐	☐	☐
10. I would provide non-pharmacological interventions.	☐	☐	☐	☐	☐	☐
11. I consider that frailty and MCI are linked	☐	☐	☐	☐	☐	☐

Item-by-item rationale
for adapting the Shanghai questionnaire to the Romanian primary care context

The instrument was adapted for the Romanian primary-care context in consultation with practising family physicians, led by a senior academic family physician (Associate Professor of Family Medicine, "Carol Davila" University of Medicine and Pharmacy, Bucharest). On the basis of this expert review, some variables were retained, others were adapted to local professional and organisational realities, and additional variables considered more relevant to Romanian primary care were introduced.

1. Practice setting

- **Original Shanghai questionnaire**: No question distinguished between rural and urban practice.
- **Romanian adapted questionnaire**: A new item was introduced asking whether the respondent's professional activity was conducted in a rural or urban setting.
- **Rationale for adaptation**: Rural–urban location was considered relevant in Romania because it may be associated with differences in access to specialists, diagnostic services, professional training and other healthcare resources. Including this variable also allowed the study to explore possible geographical inequalities in the capacity of primary care to detect and manage MCI.

2. Sex/gender

- **Original Shanghai questionnaire**: labelled "Gender," with response options Male, Female, Other.
- **Romanian adapted questionnaire**: labelled "Sex," with the same response options (Male, Female, Other) retained.
- **Rationale**: The change was terminological. The label was changed from "gender" to "sex" because the item captures a biological attribute used for demographic characterisation of the workforce rather than social identity; the response options and the underlying variable were unchanged.

4. Marital status

- **Original Shanghai questionnaire**: Married, Unmarried and Other.
- **Romanian adapted questionnaire**: Married, Not married and Other.
- **Rationale for adaptation**: "Not married" was used as a more general category that could include respondents who were single, divorced or widowed. The underlying variable was otherwise retained.

5. Working department/type of practice

- **Original Shanghai questionnaire**: The response categories were General practice, public health, Health administration and Others.
- **Romanian adapted questionnaire**: The categories referred to employment in a private practice, employment in a private medical clinic chain, ownership of a medical practice and other arrangements.
- **Rationale for adaptation**: The original categories reflected the institutional organisation of community health services in Shanghai and did not correspond directly to the organisation of Romanian family medicine. The Romanian categories were intended to capture the organisational and ownership structure of the practice, which may affect professional autonomy and access to resources.

6. Educational level

- **Original Shanghai questionnaire**: Under bachelor, Bachelor and Postgraduate.
- **Romanian adapted questionnaire**: Graduate, Postgraduate qualifications, One specialization, More specializations, PhD and Post Doc.
- **Rationale for adaptation**: The original academic categories provided limited differentiation in a sample composed of physicians, all of whom had completed university medical education. The Romanian version therefore focused on postgraduate training, additional medical specialisations and doctoral education, which were considered more relevant indicators of advanced professional preparation.

7. Professional title

- **Original Shanghai questionnaire**: Primary, Middle, Associate senior and Senior.
- **Romanian adapted questionnaire**: Primary physician, Specialist physician and No specialized fellowship.
- **Rationale for adaptation**: The professional hierarchy used in China has no direct equivalent in Romania. The categories were replaced with professional grades intended to correspond more closely to the Romanian medical training and certification system.

9. Monthly income

- **Original Shanghai questionnaire**: Included monthly after-tax income expressed in Chinese yuan.
- **Ro**manian adapted questionnaire: The income item was removed.
- **Rationale for adaptation**: The original thresholds were specific to the Chinese economic context and could not be transferred directly to Romania. Income is also a sensitive variable that may increase item non-response. It was not considered essential to the primary objective of evaluating knowledge, attitudes and practices concerning MCI, while variables such as workload, practice setting and patient-list size were more directly relevant to the organisation of primary care.

MCI detection and management context and experience

The original section contained six items. The Romanian section contained five items and was substantially adapted to reflect the workload, patient-list system and professional training context of Romanian family medicine.

1.Number of patients seen per day

- **Original Shanghai questionnaire**: <80, 80–99, 100–119, ≥120 patients per day.
- **Romanian adapted questionnaire**: <20, 20–24, 25–29, ≥30 patients per day.
- **Rationale**: The workload thresholds in the original instrument reflected the substantially higher daily patient volumes typical of Shanghai primary care and offered little discrimination in the Romanian context. On the advice of the consulted family-medicine experts, the thresholds were lowered to reflect realistic daily patient loads in Romanian family practice, so that the item would capture meaningful variation across respondents.

2. Number of patients registered on the physician's list

- **Original Shanghai questionnaire**: No corresponding item was included.

- **Romanian adapted questionnaire**: A new question assessed the number of patients registered on the physician’s patient list.
- **Rationale for adaptation**: Romanian family medicine is organised around registered patient lists. Patient-list size was therefore considered a relevant indicator of longitudinal clinical responsibility and workload. A larger list may reduce the time available for cognitive assessment, counselling and follow-up and may consequently influence the systematic detection of MCI.

3. Previous MCI-related training

- **Original Shanghai questionnaire**: A brief item asked about previous MCI training experience.
- **Romanian adapted questionnaire**: The same concept was retained, but examples were added, including MoCA certification and MMSE training.
- **Rationale for adaptation**: The examples clarified what types of training respondents should consider and reduced variation in the interpretation of the term “MCI training”. The change was intended to improve comprehensibility without substantially altering the construct.

4. MCI detection qualification versus perceived need for competence

- **Original Shanghai questionnaire**: Asked whether the respondent had an “MCI detection qualification”.
- **Romanian adapted questionnaire**: Asked whether the respondent considered it necessary to acquire specific competencies in cognitive impairment detection.
- **Rationale for adaptation**: A formal MCI detection qualification did not have a clear direct equivalent within the Romanian professional framework. The item was therefore replaced with one assessing the perceived need for further competence, which was considered more relevant for identifying continuing professional development needs in Romanian primary care.

5. Proportion of patients with memory disorders in the previous month

- **Original Shanghai questionnaire**: Asked respondents to estimate the proportion of patients with memory disorders during the previous month.
- **Romanian adapted questionnaire**: This item was removed.
- **Rationale for adaptation**: “Memory disorder” is a broad and clinically non-specific category that does not necessarily correspond to MCI. Estimating a monthly proportion may also be affected by recall error. The Romanian instrument instead included a more direct question concerning the number of patients known to have MCI.

6. Proportion of patients with psychiatric symptoms in the previous month

- **Original Shanghai questionnaire**: Asked respondents to estimate the proportion of patients with psychiatric symptoms during the previous month.
- **Romanian adapted questionnaire**: This item was removed.
- **Rationale**: In the Romanian primary-care context, psychiatric symptom assessment falls largely outside the general practitioner's formal role and follows referral pathways distinct from those for cognitive complaints. On the advice of the consulted family-medicine experts, this item was judged to lie outside the study's focus on cognitive-decline detection and to add respondent burden without contributing to the measurement of MCI-specific exposure. It was therefore removed, while the MCI-specific exposure item was retained.

7. Previous experience in MCI detection and management

- **Original Shanghai questionnaire**: Included a general Yes/No/Unsure item on previous MCI detection and management experience.
- **Romanian adapted questionnaire**: The item was removed.
- **Rationale for adaptation**: The Romanian instrument assessed relevant experience more concretely through previous training, the number of patients with MCI and the frequency of specific clinical practices. The general experience item was therefore considered potentially redundant.

8. Number of registered patients with MCI

- **Original Shanghai questionnaire**: No corresponding question was included.
- **Romanian adapted questionnaire**: A new item asked how many patients registered on the physician's list had MCI.
- **Rationale for adaptation**: The question was intended to provide a more direct measure of clinical exposure to recognised MCI cases within the physician's own practice. It also allowed clinical exposure to be considered in relation to patient-list size and reported practices.

Knowledge of general practitioners regarding MCI

The knowledge section retained eight items, but several stems and response options were modified.

K2. Modifiable risk factors affecting cognitive function

- **Original Shanghai questionnaire**: Included lack of exercise, hearing loss, depression and moderate alcohol consumption.
- **Romanian adapted questionnaire**: Retained these options and added reduced engagement in cognitively stimulating activities.
- **Rationale for adaptation**: The additional option broadened the behavioural dimension of the item and included cognitive stimulation as a potentially relevant and clinically discussable factor. It also improved consistency with the later question concerning non-pharmacological interventions.

K3. Petersen diagnostic criteria

- **Original Shanghai questionnaire**: Asked which criterion was not required for the diagnosis of MCI.
- **Romanian adapted questionnaire**: Asked which criteria were required for the diagnosis of MCI.
- **Rationale for adaptation**: The negative wording was removed to reduce the risk of misunderstanding and errors caused by overlooking the word "not". The positively worded item was intended to assess recognition of the diagnostic criteria more directly.

K4. Professionals involved in MCI diagnosis

- **Original Shanghai questionnaire**: Psychologist, psychiatrist, neurologist and All of the above.
- **Romanian adapted questionnaire**: General practitioner, psychiatrist, neurologist, geriatrician and All of the above.
- **Rationale for adaptation**: The response options were modified to reflect the clinical pathway in Romania.

K6. Treatment and management of MCI

- **Original Shanghai questionnaire**: Asked which statement was false and included the option "Donepezil should be prescribed".
- **Romanian adapted questionnaire**: Asked which statements were true, and the donepezil option was removed.
- **Rationale**: Two changes were made. First, the item's negative wording ("which statement is false") was reformulated positively ("which statements are true") to reduce avoidable cognitive load and the risk of error from respondents overlooking the word "false." Second, the donepezil option was removed because, in Romania, the prescription of anti-dementia medication is reserved for specialists (neurologists and psychiatrists) rather than general practitioners; an item testing knowledge of pharmacological treatment therefore assessed a decision outside the GP's scope of practice. The revised item focused instead on aspects of MCI management within the general practitioner's role, including the identification of reversible contributing factors and the potential for cognitive improvement.

K8. Effective non-pharmacological interventions

- **Original Shanghai questionnaire**: aerobic exercise, Mediterranean diet, music, social activities, and a donepezil option.
- **Romanian adapted questionnaire**: the donepezil option was removed; cognitively stimulating activities and an "All of the above" option were added.
- **Rationale**: Donepezil is a pharmacological agent and was conceptually inconsistent with an item explicitly assessing non-pharmacological interventions; it was removed on this basis. Cognitively stimulating activities were added as a further evidence-based behavioural intervention for cognitive health, in line with the consulted experts' input. "All of the above" was added to assess recognition that these interventions are complementary rather than mutually exclusive, consistent with a multimodal approach to cognitive risk reduction.

Attitude items

The original instrument contained 13 attitude items; the Romanian version contained 11. Most items were retained; one was reworded, one was removed, and two were merged, on the advice of the consulted family-medicine experts.

A1. MCI as part of ageing. The original statement ("MCI is not a disease, but a degenerative aging process") was changed to "…but a normal aging process." The revision was intended to express the target misconception more directly, that MCI is a normal consequence of ageing rather than a condition warranting clinical attention. Because "degenerative" and "normal" are not equivalent, this is reported as a substantive wording change that may affect the level of agreement elicited.

A11. Economic benefits (removed). The original item ("MCI detection and management provide no economic benefits") was removed because perceptions of economic benefit depend heavily on healthcare financing, reimbursement and service organisation, and the item draws on health-economic knowledge rather than clinical attitude. Its removal preserved the clinical focus of the instrument.

A12/A13 → single item (merged). Two separate items assessing whether *recognising* and *managing* MCI were GP responsibilities were combined into one: "GPs should be involved in MCI detection, diagnosis and management." The originals were partly redundant; merging them reduced respondent burden. "Should be involved" was preferred to "is the responsibility of" because MCI care spans several professional groups, so the revised item affirms a substantial GP role without implying exclusive responsibility.

Practice items
Both versions contained 11 practice items with the same frequency-based response scale. Several items were reworded, one removed, one replaced, and one newly introduced, on the experts' advice.

P3. Family history of Alzheimer's disease. Wording changed only in verb tense ("has" → "had"); no change in content. (This is an English-grammar edit, not an adaptation, see note below.)

P4. Detection of MCI risk factors. "I would detect MCI risk factors" became "I think that it is important to detect MCI risk factors," capturing the importance the physician assigns to risk-factor identification.

P5. Use of MCI screening methods. The hypothetical "I would utilise…" was changed to the behavioural "I use the MCI screening methods during patient visits," converting the item from intention to current reported behaviour and specifying the context, more appropriate for a frequency-based scale.

P6. Specialist involvement in diagnosis. "I would get specialist advice for final diagnosis by transference" became "I would consult with specialists for final diagnosis by transference," expressing consultation and referral more clearly.

P9. Coordination of support services (removed). Removed because the organisation and meaning of "support services" vary across health systems and do not correspond to a clearly defined element of Romanian primary care, risking inconsistent interpretation.

P10 → Romanian P9. Prescribing medication. "I would prescribe medications" became "I would prescribe medications recommended by specialists," clarifying that the item concerns treatment continuity and implementation of specialist recommendations rather than independent initiation of anti-dementia medication, which, in Romania, is reserved for specialists. This reflects the actual Romanian clinical pathway.

P11 → Romanian P10. Non-pharmacological interventions. Retained unchanged; renumbered from 11 to 10.

Romanian P11. Frailty and MCI (new). A new item was added, "I consider that frailty and MCI are linked", to explore whether GPs recognise the relationship between cognitive and physical vulnerability in older adults, reflecting an integrated, multidimensional approach to ageing.
